# An Investigation of the High Performance of a Novel Type of Benzobisoxazole Fiber Based on 3,3-Diaminobenzidine

**DOI:** 10.3390/polym8120420

**Published:** 2016-12-13

**Authors:** Yang Wang, Yuanjun Song, Lei Zhao, Nahla Rahoui, Bo Jiang, Yudong Huang

**Affiliations:** 1 MIIT Key Laboratory of Critical Materials Technology for New Energy Conversion and Storage, School of Chemistry and Chemical Engineering, State Key Laboratory of Urban Water Resource and Environment, Harbin Institute of Technology, Harbin 150001, China; hitwangyang@hit.edu.cn (Y.W.); songyj@hit.edu.cn (Y.S.); zhaolei328@hit.edu.cn (L.Z.); nahlarahoui@hit.edu.cn (N.R.); 2 College of Chemistry, Chemical Engineering and Materials Science, Heilongjiang University, Harbin 150001, China

**Keywords:** third comonomer, benzobisoxazole fibers, high performance

## Abstract

The mechanical and thermal properties of poly{2,6-diimidazo[4,5-b:4′5′-e] pyridinylene-1,4(2,5-dihydroxy) phenylene} (PIPD)-3,3-diaminobenzidine (DAB) fibers were analyzed. Compared to other types of benzimidazole fiber structures and properties, PIPD-DAB is distinguished by a unique combination of strength, tensile modulus, and thermal properties. The PIPD polymer was prepared from 2,3,5,6-tetra-aminopyridine (TAP) and 2,5-dihydroxyterephthalic acid (DHTA) in polyphosphoric acid (PPA). In order to enhance the tensile strength and modulus, a third comonomer, 3,3-diaminobenzidine (DAB), was incorporated into the PIPD molecular structure. The change in molecular structure was recorded using Fourier-transform infrared (FT-IR) spectroscopy, nuclear magnetic resonance (NMR) spectroscopy, and wide angle X-ray diffraction (WAXD). Compared to the PIPD fibers (average tensile strength of PIPD is 3.9 GPa, average tensile modulus of PIPD is 287 GPa), the tensile strength and modulus of PIPD-DAB increased to 4.2 and 318 GPa, respectively. In addition, the thermal decomposition temperature of the PIPD fibers is enhanced by 35 °C, due to the incorporated DAB. PIPD-DAB is a promising material for use under high tensile loads and/or high temperatures.

## 1. Introduction

High performance fibers, with elevated mechanical and thermal properties, have become important in modern industry, transport, sports, and other areas that create high-strength composites, highly loaded textile structures, and thermostable and difficult-to-combust articles. PIPD fibers, which are representative of high-performance fibers, are characterized by their high-strength, high-modulus, and high thermal stability [1,2,3,4,5]. However, even for the high-modulus PIPD fibers developed by Sikkema [6] and co-workers, a large gap between the actual tensile modulus (330 GPa) [7] and the theoretical tensile modulus remains (the chain modulus of PIPD calculated with Gaussian 09 is as high as 543–560 GPa) [8,9]. If impurities, voids, and entanglements are eliminated, this would result in a continuous fiber with a perfectly oriented structure, low density, and a tensile strength close to that of the theoretical value [10]. A perfectly oriented structure could leads to a distinctive feature within extremal mechanical properties with high loaded textile items and high strength, which are distinctive features of these high-strength and high-modulus fibers. To obtain perfectly oriented reinforced fibers with maximum mechanical properties, it is necessary to ensure the maximum energy of the interatomic bonds in the polymer chains (layers), the absence of “weak” bonds, a maximum regularity of the chain structure, the degree of polymerization, and supramolecular ordering. High-strength and high-modulus fibers are manufactured from different kinds of aromatic, and some aliphatic, polymers. High-modulus para-aramid fibers are made from heterocyclic *p*-polyamides and similar copolyamides (SVM, Rusar and Armos, Moscow, Russia), with a dynamic deformation modulus of 120 to 160 GPa. SVM fibers (historically, the first kind of high-modulus heterocyclic aromatic fibers) are based on polyamidobenzimidazole, which is synthesized from heterocyclic *p*-diamine and terephthalochloride. The copolymeric heterocyclic para-aromatic fibers Armos and Rusar are based on the same components and incorporate *p*-phenylenediamine as a third comonomer [11]. The present experience in the area of poly-heterocyclic-para-aramides (SVM, Rusar and Armos, Moscow, Russia) shows better results when the initial solution is non-liquid-crystalline (isotropic) and the transition to the liquid-crystalline state occurs during fiber structure formation. The rapid crystallization of regular para-aramides can prevent the formation of maximum orienting order due to the decrease in molecular mobility, both in the fiber structure formation stage (by spinning) and in thermal treatment [12]. The same situation is noted in the cases of semi-rigid polymer–cellulose and polyacrylonitrile in organic solutions. The limitations in the structuring and crystallization of these solutions during fiber formation result in a higher orientation and mechanical properties of the fibers [11].

The PIPD fiber is based on 2-, 3-, 5- and 6-tetramine pyridine hydrochloride (TAP) and 2,5-dihydroxyterephthalic acid (DHTA) [7,13]. Because the hydrogen bonding network is crucial for the tensile modulus and strength [14], the presence of numerous hydroxyl groups at the edges of DHTA implies that PIPD could interact strongly with other polar polymer matrices. This strong interaction, combined with its high surface area and high modulus, qualifies its application in reinforcing other polymer matrices [15,16]. The unique feature of these polymers is that the two hydroxyl groups (in terephthalic acid) can form intermolecular hydrogen bonds; therefore, fibrillation, which is often a problem for aramid fibers and PBO fibers, is practically eliminated. As a result, PIPD fibers have the highest compressive strength among synthetic fibers [7]. However, PIPD fibers do not have the highest tensile modulus and tensile strength among synthetic fibers.

**Figure 1:**
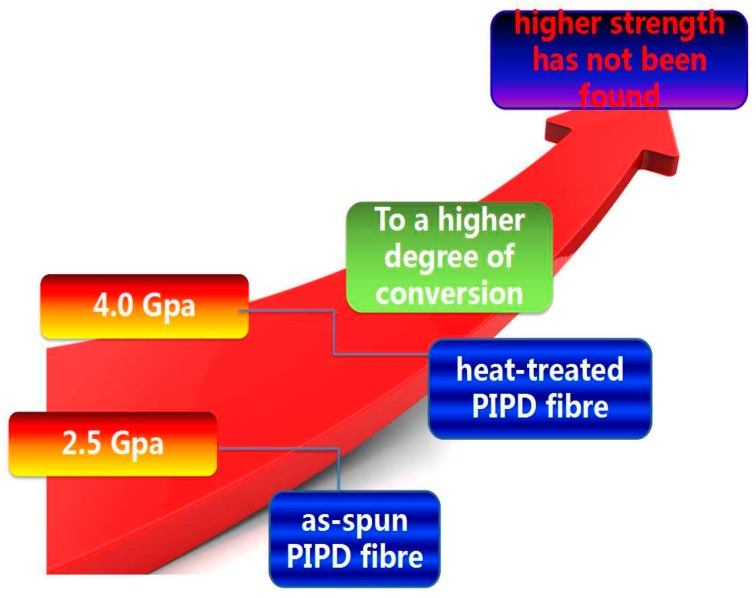
Sketch of poly{2,6-diimidazo[4,5-b:4′5′-e] pyridinylene-1,4(2,5-dihydroxy) phenylene} (PIPD) fiber strength.

3,3-diaminobenzidine (DAB) was initially used to produce high-strength polybenzimidazole fibers, as reported in U.S. Patent No. 3987015 [17]. In this study, a third comonomer, 3,3-diaminobenzidine, is incorporated into the PIPD molecular structure. Because π bonding is important for modulus and strength, the presence of the two phenyl groups in DAB implies that PIPD-DAB has a more interatomic bond energy, a greater number of transfer and load-holding chains in polymer chains (layers), and an absence of “weak” bond side substituents and branches. The additional incorporation of a third type of links (DAB) in the molecular chains results in partially statistical irregularity. Therefore, PIPD-DAB cannot entirely form a liquid-crystalline order in solutions, nor a 3D crystalline order in the solid state. PIPD-DAB is fundamentally different in comparison with unmodified PIPD, which is liquid-crystalline in solutions and 3D crystalline in a solid state. It follows from the above that the rapid crystallization of regular PIPD may prevent the maximum orienting order due to the decrease in molecular mobility, both in the stage of the fiber structure formation and in thermal treatment. The partial liquid–crystalline order in the solutions during fiber formation caused more approaches in the direction of most bonds to the direction of the chain axis, a less effective chain cross section, and more degrees of polymerization and of supramolecular ordering (crystallinity, orientation). Figure 1 shows the PIPD fiber tensile strength. Up to now, prepared PIPD fiber moduli have been 150–360 GPa, and tensile strengths have been 2.5–4.0 GPa [6,18,19,20,21]. To the best of our knowledge, no experimental study has been conducted in order to improve the tensile strength and heat resistance of PIPD fibers using the incorporation of a third comonomer.

An alternative method to increase the tensile strength of PIPD fibers is the incorporation intermolecular hydrogen bonds [22,23]. To obtain a higher orientation of the polymer chain with better mechanical properties, we report here on a new approach in the preparation of PIPD-DAB copolymers using one-pot, in situ polycondensation in acidic media.

**Figure 2:**
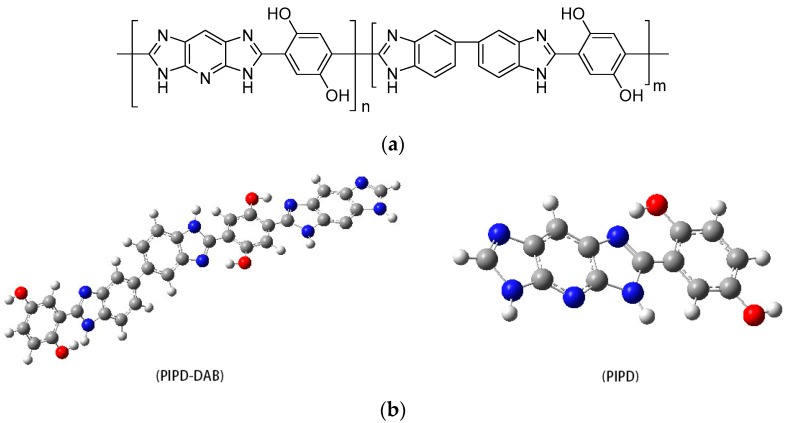
(**a**) Chemical structure of modified polybenzimidazole poly PIPD-3,3-diaminobenzidine (DAB); (**b**) structural formula of polybenzimidazole poly PIPD and PIPD-DAB (gray: C, blue: N, red: O, white: H).

The novel polybenzimidazole polymer fiber (PIPD-DAB) is a high performance organic polymer fiber; the polymer’s repeating unit is illustrated in Figure 2. A third comonomer with a distorted structure enhances the tensile and modulus strengths, which will have a considerable effect on the development of high-performance organic fibers. Schematic diagrams of the synthesis of PIPD-DAB are shown in the Supplementary Materials Scheme S1.

## 2. Experimental Section

### 2.1. Materials

The comonomer, 2,5-dihydroxyterephthalic acid (DHTA), was obtained from dimethyl 1,4-cyclohexanedione-2,5-dicarboxylate (DMSS) under the action of hydrogen peroxide, with potassium iodide as catalyst, to obtain an intermediate, 1,4-hydroquinone-2,5-dicarboxylate (DMDHT) and then, under alkaline conditions using hydrolysis. DAB was synthesized from 3,3′-dinitrobenzidine (DNB) using the general technique described in U.S. Patent No. 3943175 [24] and U.S. Patent No. 7999112 [25]. TAP was obtained from 2,6-diaminopyridine using a typical method [26], and DNB and polyphosphoric acid (PPA) were purchased from the Beijing Reagents Company (Beijing, China) and were used as received.

### 2.2. Copolymerization of PIPD-DAB

A mixture of 75.0 g of 85% orthophosphoric acid and 175.0 g of PPA (84% P_2_O_5_ content) was stirred under a reduced pressure for 2 h at 100 °C. After cooling to room temperature, a mixture of TAP and DAB (0.23 mol) was added to the obtained PPA. Then, 0.23 mol of freshly dried DHTA was added to the above-mentioned solution in six portions.

After the addition of each portion of the monomer, stirring is initiated. The mixture was then stirred while the temperature was increased slowly and the pressure was decreased slowly until dehydrochlorination was complete. P_2_O_5_ (87.5 g) was then added to the dehydrochlorination mixture at 45 °C. The mixture was then stirred for 2 h at 95 °C. The polymerization was then stirred under a nitrogen atmosphere, at 160 °C, for approximately 10 h, at 180 °C for approximately 3 h, and at 200 °C for 3 h. The green product exhibited a metallic luster.

### 2.3. Spinning Technology

**Figure 3:**
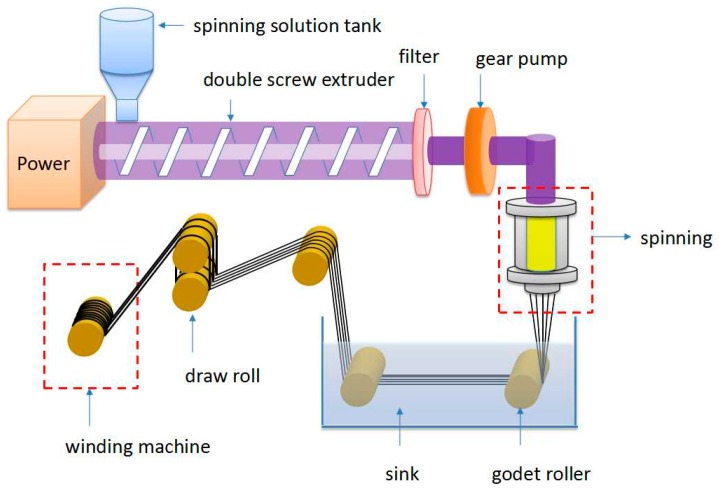
PIPD fibers spinning apparatus schematic.

A schematic drawing of the spinning technique is shown in Figure 3. The double screw extruder conditions were as follows: diameter, 25 mm; aspect ratio, 20; all zone temperature, 180 °C; screw speed, 5 rpm. A spinning dope, which contained the copolymer of PIPD-DAB (with an inherent viscosity of approximately 21 dL/g, measured at 25 °C) that was extruded from 49 orifices spinneret at 180 °C, was used. Each orifice diameter at the exit point was 0.23 mm. The filaments were drawn over a coagulation bath containing deionized water at room temperature and were located 35 cm from the spinneret. The velocity of the filaments entering the coagulation bath was 150 m/min. After being coagulated, the filaments were washed, dried, and wound on a spool.

### 2.4. Characterizations and Measurements

A Fourier-transform infrared (FT-IR) spectrometer (Nicolet-Nexus 670, Nicolet Instrument. Inc., Madison, AL, USA) was used for characterization of chemical structure. Raman spectra were collected at 780 nm Ar^+^ ion excitation on a Renishaw InVia Reflex Raman spectrometer (Renishaw plc, Gloucestershire, UK) in a backscattering configuration. Wide angle X-ray diffraction (WAXD) experiments were conducted on an X-ray generator (Rigaku Corporation, Tokyo, Japan) using Cu Kα radiation (0.1542 nm). Solid-state ^13^C cross-polarization magic-angle-spinning nuclear resonance (^13^C CPMAS NMR, Bruker Daltonics Inc., Karlsruhe, Germany) experiments were performed at 9.4 T at a Varian frequency of 100.4 MHz. PIPD and PIPD-DAB fibers were examined using a scanning electron microscope (Netherlands Philips FEI Sirion, Field Electron and Ion Co., Hillsboro, OR, USA) operated at 10 kV using a calibration specimen. Energy-dispersive X-ray spectroscopy (EDX, EDAX Inc., Mahwah, NJ, USA) was used for elemental analysis and characterization of surface roughness. Thermogravimetric analysis (TGA) measurements were carried out on a Mettler Toledo 851e thermogravimetric analyzer (German NETZSCH Instruments Manufacturing Co., Ltd., Bavaria, Germany). Samples were heated from room temperature to 1000 °C in a dynamic nitrogen environment at heating rates of 5, 10, 20 and 50 °C·min^−1^. Single-fiber tensile testing was performed on a universal tensile tester (model WD-1, Shenzhen Sans Testing Machine Co. Ltd., Shenzhen, China) instrument. The length of the fiber samples was set at 30 mm. For each fiber, at least 50 monofilaments were tested and the average values were considered as representative.

## 3. Results and Discussion

### 3.1. Synthesis and Characterization of DAB

**Figure 4:**
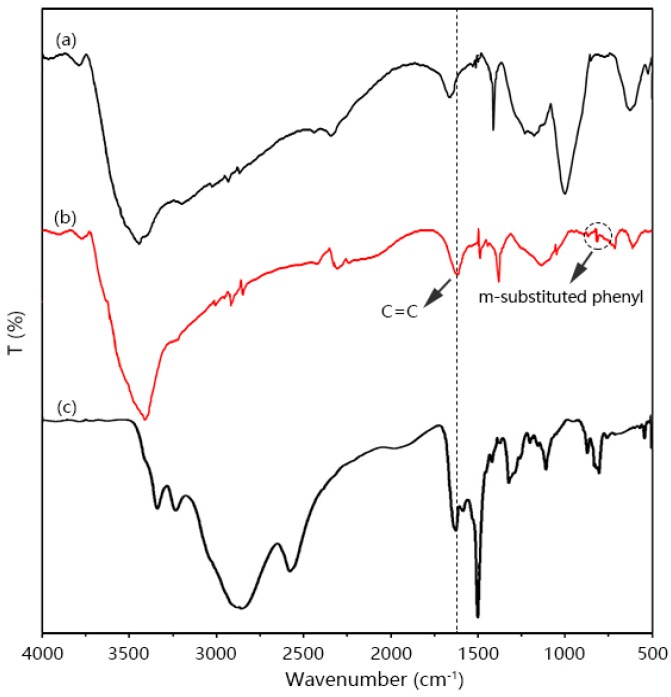
Fourier-transform infrared (FT-IR) spectra of (**a**) PIPD; (**b**) PIPD-DAB; and (**c**) DAB.

DAB has a symmetrical structure within a central carbon bond between two rings. Figure 4c shows the FT-IR spectra of DAB. The FI-IR spectra show all arising DAB corresponding resonances. As can be observed in the FTIR spectra of DAB, peaks at 3400–3200 cm^−1^ are assigned to –NH_2_ stretching modes, and 1625 cm^−1^ corresponds to the C=C graphitic stretching of the aromatic ring. The peaks at 1370 cm^−1^ are assigned to the C–N stretch. The ^1^H-NMR spectra and Raman spectra of DAB are shown in the Supplementary Materials Figures S1 and S2.

### 3.2. Structural Characterization of PIPD-DAB Copolymers

Figure 4 shows the FT-IR spectra of PIPD and PIPD-DAB. Figure 4a,b show a wide band centered at 3400 cm^−1^, corresponding to N–H and O–H stretching vibrations. All the spectra present characteristic peaks of PIPD and PIPD-DAB; absorption peaks at ~1380 cm^−1^ can be assigned to the C–N stretching between imidazole and benzol [27]. The peaks at ~1625 cm^−1^ can be assigned to the C=C stretching of the pyridine in PIPD-DAB. In addition, the FT-IR spectra of PIPD-DAB are notably similar to the PIPD spectrum. According to peak characteristics in Figure 4b, the peaks at ~810 cm^−1^ can be assigned to the m-substituted phenyl of DAB, which confirms that DAB was successfully incorporated into the polymer PIPD.

**Figure 5:**
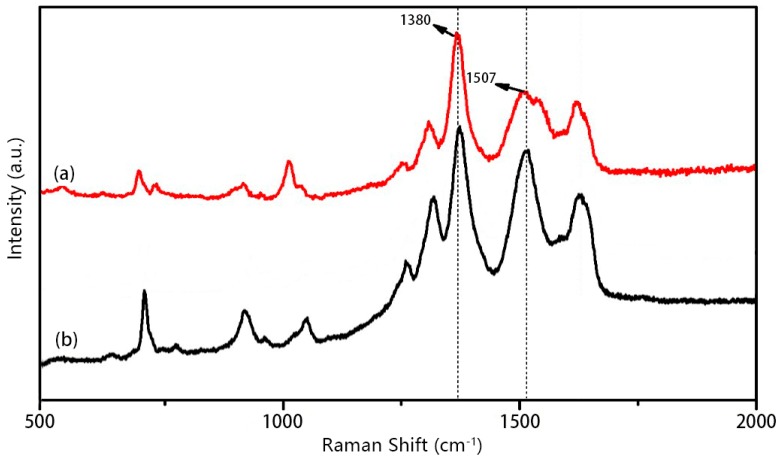
Typical Raman spectra obtained from a single filament of (**a**) PIPD and (**b**) PIPD-DAB.

A typical Raman spectrum for the PIPD-DAB fiber, in the region of 500–2000 cm^−1^, is shown in Figure 5. The spectrum consists of a few well-defined and intense peaks on a strong fluorescent background. Three intense Raman bands on the fluorescent background, in the region of 1300–1800 cm^−1^, clearly appear in the Raman spectra of the PIPD and PIPD-DAB fibers. The most intense bands are situated at 1380 cm^−1^, which are principally assigned to C=C stretching. The 1507 cm^−1^ band can be assigned to C=N stretching vibrations. The Raman spectrum shows that the peak intensity at 1507 cm^−1^ for PIPD-DAB is stronger than that of PIPD. This might be attributed to the presence of DAB after copolymerization. These structure characterization results confirmed that DAB was successfully incorporated into the polymer PIPD.

### 3.3. Characterization and Properties of PIPD-DAB Copolymer Fibers

**Figure 6:**
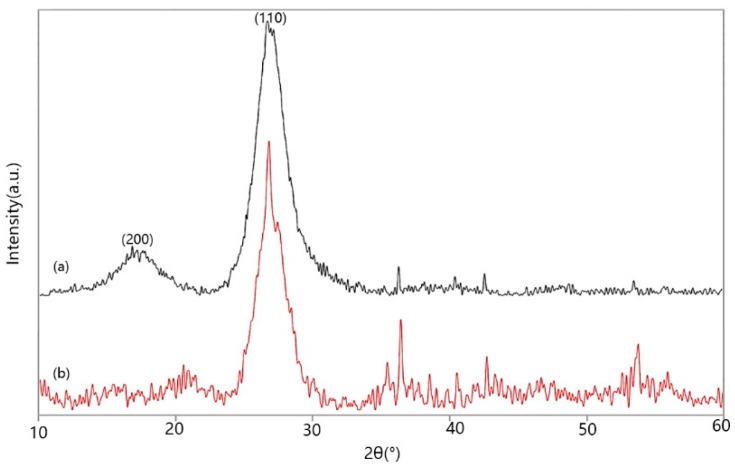
Wide-angle X-ray diffraction (WAXD) pattern of (**a**) PIPD and (**b**) PIPD-DAB.

WAXD patterns from equatorial scans of PIPD and PIPD-DAB fibers are shown in Figure 6. In Figure 6a, two diffraction peaks at (200) and (110) can be observed due to PIPD being used as the liquid crystal polymer. As revealed in Figure 6b, the (110) peaks can be observed; the (200) peaks gradually disappeared. This may have been caused by the transition of states, from a liquid crystalline state to a non-liquid crystalline state. In addition, the polymer chain orientation of PIPD and PIPD-DAB could be computed using Herman’s orientation factor (Equation (1)) [28]. Herman’s orientation factor equation can be simplified as Equation (2).

(1) $$f=\frac{3\langle \cos^{2}\varphi \rangle -1}{2}$$

(2) $$\pi =\frac{180{}^{\circ}-H}{180{}^{\circ}}\times 100\%$$

where π is the polymer chain orientation, and *H* is the half-width of the reflection at a scattering angle of 2θ. The polymer chain orientation is obtained using Equation (2) from the half-width of the diffraction peaks (110) at 27.08°. Through analyses using JADE 6.0 software (Materials Data, Inc., Livermore, CA, USA), *H*_PIPD-DAB_ = 2.478°, and *H*_PIPD_ = 2.778°. After calculation, π_PIPD-DAB_ = 98.62%, π_PIPD_ = 98.45%, and π_PIPD-DAB_ > π_PIPD_. This indicates that PIPD-DAB has a higher polymer chain orientation than PIPD. In summary, a proper amount of DAB was able to improve the polymer chain orientation via control of the transition state, from a liquid crystalline state to a non-liquid crystalline state, which is consistent with the conclusions of [12].

**Figure 7:**
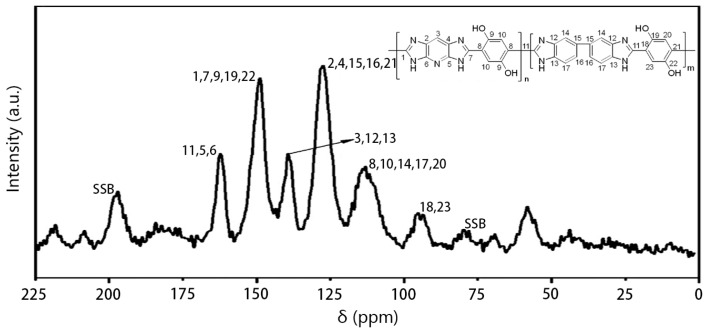
Solid state ^13^C NMR spectra of PIPD-DAB copolymers. SSB: Spinning Side Band.

NMR is counted as a useful tool for providing valuable information on covalently functionalized PIPD-DAB copolymers. Figure 7 reveals the ^13^C NMR spectra of PIPD-DAB. The ^13^C NMR spectrum of PIPD-DAB shows a characteristic resonance at 139 ppm, which confirms the formation of benzimidazole rings. Furthermore, the characteristic resonance at 127 ppm and a new resonance at 115 ppm, which further confirms that DAB was successfully incorporated into the polymer PIPD. The ^13^C NMR spectra of functionalized PIPD-DAB copolymers were well characterized in order to confirm the formation of a covalent bond between PIPD and DAB.

**Figure 8:**
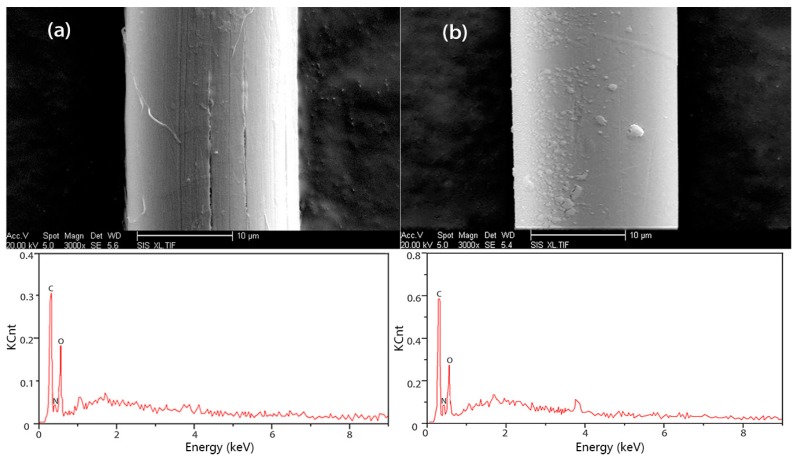
Representative SEM and Energy Dispersive X-ray Spectroscopy (EDX) images of (**a**) PIPD and (**b**) PIPD-DAB.

As shown in Figure 8, the compositions of PIPD and PIPD-DAB are characterized using Energy Dispersive X-ray Spectroscopy (EDX) and SEM images. Figure 8 demonstrates the morphology of the longitudinal outer surfaces of PIPD and PIPD-DAB fibers. It is well known that PIPD fibers consist of fibril bundles of 20 μm and fibrils of a smaller size. This fiber structure can be partially seen in Figure 8a. There are slight cracks and micro-fibrils on the fiber surfaces, which may be caused by the friction between the fiber spinneret and fiber scoops in the spinning process [29]. The surface texture also reflects the uniformity of the coagulation process, and subsequent fiber shrinkage is due to the volume loss of the solvent PPA. It is evident from Figure 8a,b that the outer surface of the PIPD-DAB fiber is smoother than that of the PIPD fiber. The reason for this may be associated to the incorporation of DAB into the PIPD chains, which can enhance the tenacity of the fiber upon a beneficial improvement in the mechanical properties of the fibers.

EDX proved to be a powerful tool for chemical analysis. After DAB incorporation in PIPD, the carbon content was raised from 55.87% to 62.23% (theoretical computations were performed; carbon content was raised from 58.86% to 70.58%), but the oxygen content diminished from 11.75% to 10.11% (theoretical computations were performed; oxygen content decreased from 12.08% to 9.41%), and the nitrogen content decreased from 26.27% to 20.74% (theoretical computations were performed; nitrogen content decreased from 26.41% to 16.47%). There are some deviations between the test results and the theoretical results—probably caused by the fact that the phosphates in the synthesized polymer were difficult to eliminate.

**Figure 9:**
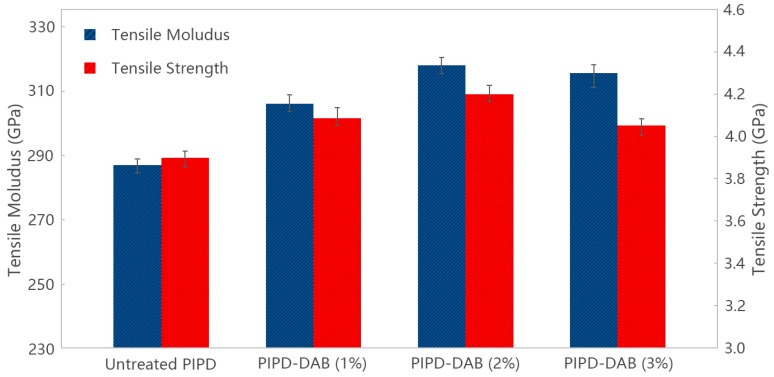
Mechanical properties of PIPD and PIPD-DAB.

Figure 9 demonstrates that the mechanical properties of PIPD and PIPD-DAB polymer fibers. PIPD fibers present an average tensile strength of 3.9 GPa, and an average tensile modulus of 287 GPa. The heat-treated fiber tensile strength and modulus, compared to unmodified PIPD fibers after the addition of 1% DAB, increased by 4.8% and 6.7%, respectively, while those of PIPD-DAB (2%) are 7.7% and 10.8% higher than the values for the PIPD fibers, respectively. However, the mechanical characteristics and tensile strength, tensile modulus, and strain to failure of PIPD-DAB (3%) fibers decreased by 3.9% and 10.0%, respectively. The enhanced mechanical properties should be attributed to DAB, which is indicative of the fact that the rapid crystallization of regular PIPD may prevent reaching the maximum orienting order due to the decrease in molecular mobility, in the fiber structure formation stage (by spinning) and in thermal treatment. When the concentration of PIPD-DAB reached 1% and 2%, the solution presented a non-liquid-crystalline (isotropic) state and then a change in transition to the liquid-crystalline state occurs during the process of fiber structure formation, which contributes to an improvement in mechanical properties. However, the excess addition of PIPD-DAB, e.g., a content of 3% may make the transition state, as mentioned above, a non-liquid-crystalline (isotropic) state, which consequently contributes to a decline in tensile strength and modulus.

**Figure 10:**
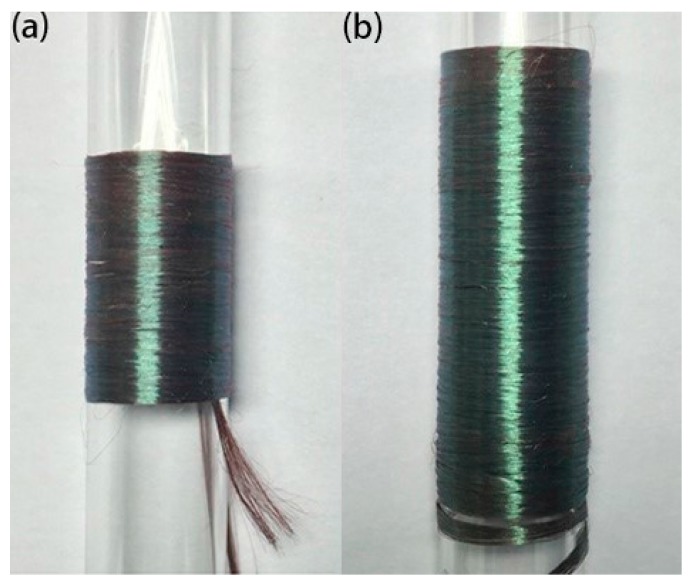
Photograph of (**a**) PIPD and (**b**) PIPD-DAB (2%) fibers collected using spools.

Photographs of the copolymer and PIPD fibers are shown in Figure 10. The copolymerization of DAB with PIPD was successfully carried out using a dry-jet wet-spinning technique. When the solvent and the temperature were constant, the molecular weight of PIPD was related to the intrinsic viscosity (η). The weight-average molecular weight was calculated from the intrinsic viscosity using the equation, [η]/dL·g^−1^ = 2.27 × 10^−7^ × *M*_w_^1.8^. The intrinsic viscosities (η) for PIPD and PIPD-DAB (2%) were 15.4 and 20.5 dL/g, respectively. The average molecular weights of PIPD, PIPD-DAB were calculated to be 2.24 × 10^4^ and 2.59 × 10^4^ g/mol, respectively. The PIPD-DAB copolymer has a higher molecular weight, which might be related to the third comonomer. During the polymerization reaction, the introduction of DAB, with a flexible molecular structure, was beneficial in decreasing the viscosity and increasing the fluidity, which contributed to a better polymerization within the enhancement of molecular weight and intrinsic viscosity. The mechanical property results improved significantly, while the molecular weight increased slightly, as shown in Figure 10, which means that the transition state is useful for the copolymer to obtain a better molecular orientation.

### 3.4. Thermal Degradation Reaction Kinetics of PIPD Fiber Based on DAB

**Figure 11:**
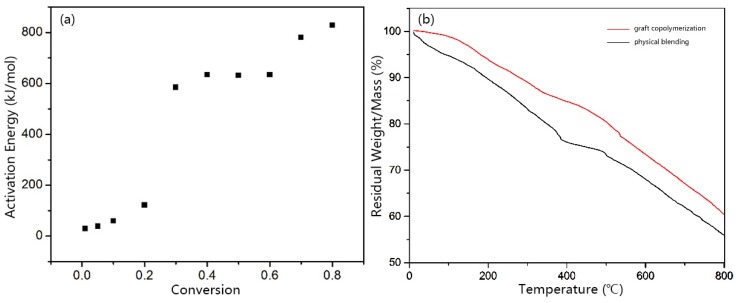
(**a**) The activation energies of PIPD-DAB at different conversions; (**b**) The curves of graft copolymerization and physical blending composite fibers.

The process of PIPD fiber thermal degradation is shown in the Supplementary Materials Figures S3–S7. As illustrated in Figure 11a, the thermal decomposition activation energy of DAB-modified PIPD also showed two platforms within a weight loss α ranging from 0.01 to 0.6, and an average activation energy of 212.53 kJ/mol, for the first platform, and a weight loss α ranging from 0.7 to 0.9, and an average activation energy of 713.39 kJ/mol, for the second platform. The third comonomer, DAB, was added in order to improve the thermal decomposition activation energy by way of an improvement in the thermal stability of the PIPD fibers. In general, the activation energy of thermal decomposition has an increasing trend, with an increase in the degree of decomposition, which indicated that weight loss becomes progressively more difficult [30]. Due to the initial phase of the thermal degradation reactions, small molecules were degraded; the decomposition reaction activation energy is notably lower with an increase in the PIPD-DAB weight loss rate. The required reaction temperature is higher when the conversion increased to 0.3; macromolecular compounds were degraded, decomposition reaction energy then increased, and the reaction activation increased significantly. 

The TG of the graft copolymerization and the physical blending of composite fibers are shown in Figure 11b; the derivative weight loss of TG is shown in the Supplementary Materials Figure S8. The thermal decomposition temperature of graft copolymerization is shown to be 535 °C; the thermal decomposition process presents only one phase. For the physical blending of PIPD-DAB fibers, a TG curve provides the decomposition of two phases. In the first phase, DAB molecule decomposition is shown to be 385 °C; then, in the second phase, the decomposition of the PIPD structure is shown to be 500 °C (Supplementary Materials Figure S8). The third comonomer is incorporated into the PIPD structure via graft copolymerization; thermal decomposition of the polymer was enhanced by 35 °C. This enhancement was caused by the strong chemical bond–π bond in DAB molecules.

## 4. Conclusions

In this study, we successfully carried out the copolymerization of DAB with PIPD via a simple and efficient method. A proper amount of the third comonomer could improve the molecular orientation by controlling the transition state, from a liquid crystalline state to a non-liquid crystalline state, and improve the tensile modulus and tensile strength of PIPD-DAB composite fibers by 10.8% and 7.7%, respectively, with respect to PIPD fibers prepared under the same conditions. The addition of the third comonomer is not only suitable for enhancing the mechanical properties of PIPD, but can also be used as a novel process to improve the properties of other polymer fibers.

## Supplementary Materials

The following are available online at www.mdpi.com/2073-4360/8/12/420/s1, Scheme S1: Schematic diagrams for the synthesis of PIPD-DAB; Figure S1: ^1^H-NMR spectra of the DAB; Figure S2: Raman spectra of the DAB, Figure S3: TG curves of PIPD fibers at different heating rates; Figure S4: PIPD fibers curves between the conversion and the temperature at different heating rates; Figure S5: TG curves of PIPD-DAB fibers at different heating rates; Figure S6: PIPD-DAB fibers curves between the conversion and temperature at different heating rates; Figure S7: The relationship curve of logβ and 1/T; Figure S8: The derivative weight loss of (a) graft copolymerization, (b) physical blending composite fibers.
