# Supplementary material for: An Investigation of the High Performance of a Novel Type of Benzobisoxazole Fiber Based on 3,3-Diaminobenzidine
Source: Polymers (Basel). 2016 Dec 13;8(12):420. doi: 10.3390/polym8120420 (PMC6432024; doi:10.3390/polym8120420)
Supplement: Supplementary file 1 [file polymers-08-00420-s001.pdf]

Supplementary Materials: An Investigation of the High Performance of a Novel Type of Benzobisoxazole Fiber Based on 3,3-Diaminobenzidine

Yang Wang, Yuanjun Song, Lei Zhao, Nahla Rahoui, Bo Jiang and Yudong Huang

1. Synthesis of PIPD-DAB

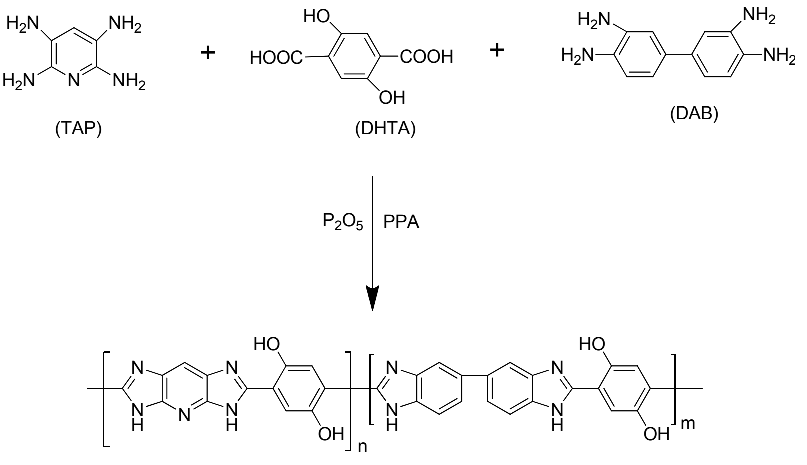

Scheme S1. Schematic diagrams for the synthesis of PIPD-DAB.

2. Characterization of DAB Monomer

In this work, DAB was well characterized by ^{1}H-NMR and Raman. The results confirm the chemical structure of DAB. In the spectrum of DAB, the ^{1}H-NMR (Figure S1) spectrum shows all the corresponding resonances arising from DAB. Raman spectra of the DAB is given in Figure S2.

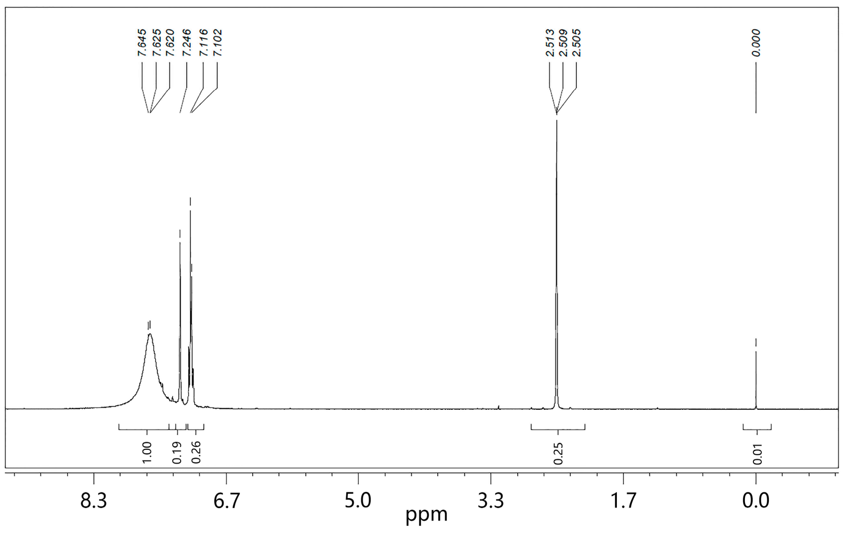

Figure S1. ^{1}H-NMR spectra of the DAB.

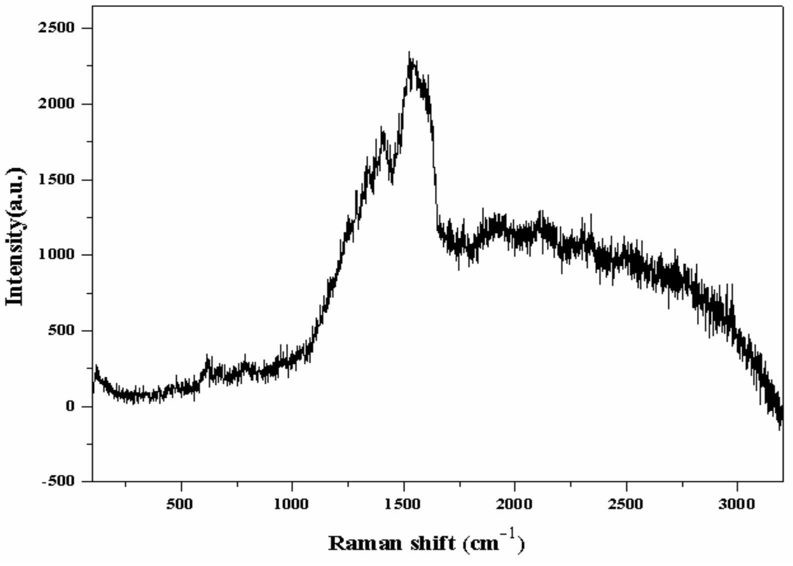

Figure S2. Raman spectra of the DAB.

3. Thermal Degradation Reaction Kinetics of PIPD Fiber Based on DAB

When the thermal decomposition reaction kinetics parameters are calculated by the method of heat loss, the decomposition degree (conversion) of the polymer can be calculated by the following formula:

$$\alpha = \frac{(M_0 - M_t)}{(M_0 - M_f)} \tag{S1}$$

where, M_0, M_T and M_f are the initial mass, the practical mass, and the final mass after degradation, respectively.

After the separation of variables and then the integral of the previous formula, we can be obtain:

$$\log\beta = \log\frac{AE}{RF(\alpha)} - 2.315 - \frac{0.4567E}{RT} \tag{S2}$$

It is obvious from the Equation (2) that, when the degree of decomposition is a fixed value, the value of $F(\alpha)$ is constant. The plot of $\log\beta \sim 1/T$ is a straight line at different heating rates. The thermal decomposition activation energy could be obtained from the plot slope $-0.4567E/R$ as illustrated in the Equation (3). Ozawa TGA curve method requires at least four different heating rates to determine the thermal decomposition activation energy.

$$E = -k \times \frac{R}{0.4567} \tag{S3}$$

Ozawa method requires a number of different heating rates of TG curve to solve the thermal decomposition activation energy equation. Figures S3 and S5 is TG curves of PIPD and PIPD-DAB fibers within different heating rate: 5, 10, 20 and 50 °C/min, respectively. Figures S4 and S6 show the relationship between the logarithm of heating rate and the reciprocal of absolute temperature for the PIPD and PIPD-DAB under different degree of decomposition.

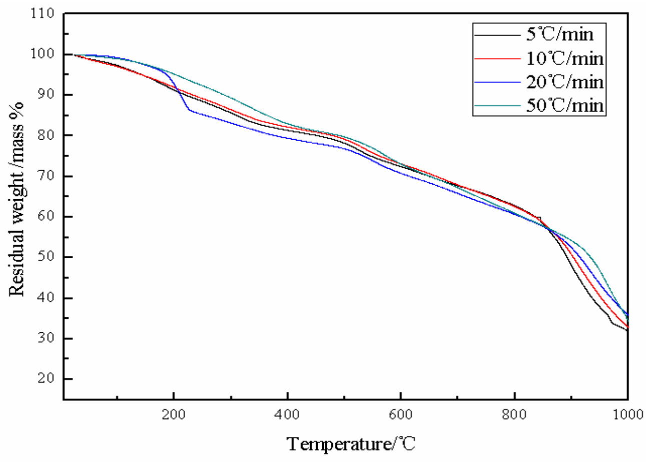

Figure S3. TG curves of PIPD fibers at different heating rates.

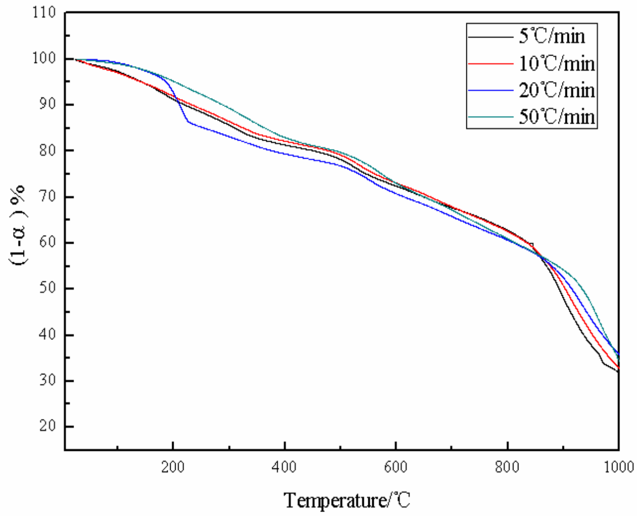

Figure S4. PIPD fibers curves between the conversion and the temperature at different heating rates.

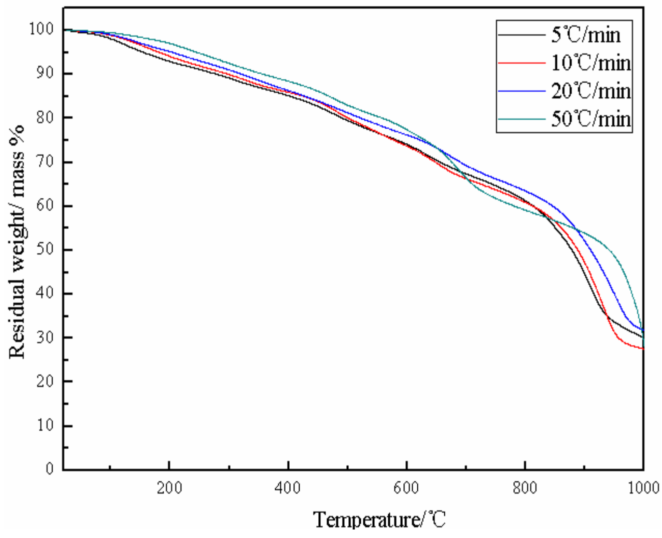

Figure S5. TG curves of PIPD-DAB fibers at different heating rates.

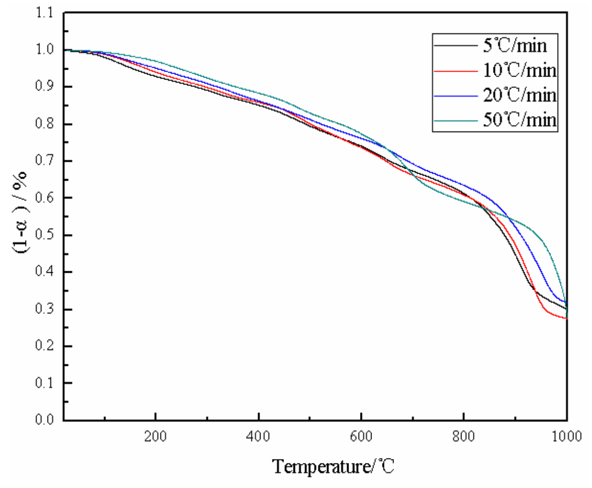

Figure S6. PIPD-DAB fibers curves between the conversion and temperature at different heating rates.

Figure S5 shows the TG curves of PIPD-DAB fibres at heating rates of 5, 10, 20 and 50 °C/min respectively. Figure S6 reveals the relationship between the logarithm of temperature rising rate and the reciprocal of absolute temperature under different decomposition degree of PIPD-DAB. The apparent activation energy is calculated according to the Equation (3). The pyrolysis activation energy at different decomposition degree is illustrated in Figure S7.

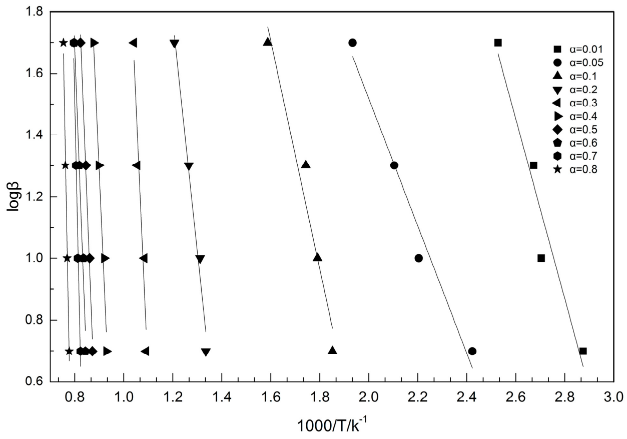

Figure S7. The relationship curve of logβ and 1/T.

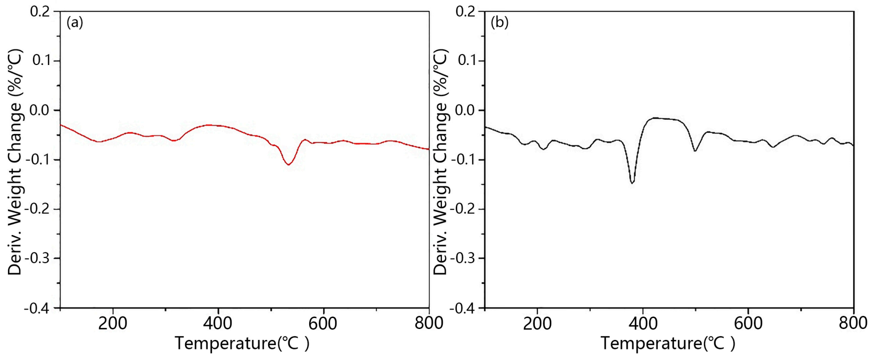

Figure S8. The derivative weight loss of (**a**) graft copolymerization; (**b**) physical blending composite fibers.
